# Senescence-related gene c-Myc affects bladder cancer cell senescence by interacting with HSP90B1 to regulate cisplatin sensitivity

**DOI:** 10.18632/aging.204863

**Published:** 2023-07-10

**Authors:** Yaxuan Wang, Haixia Zhu, Haifei Xu, Yifan Qiu, Yonghong Zhu, Xiaolin Wang

**Affiliations:** 1 Department of Urology, Affiliated Tumor Hospital of Nantong University and Nantong Tumor Hospital, Nantong 226361, China; 2 Department of Central Laboratory, Affiliated Tumor Hospital of Nantong University and Nantong Tumor Hospital, Nantong 226361, China; 3 Department of Urology, The First Affiliated Hospital of Soochow University, Suzhou 215006, China

**Keywords:** HSP90B1, c-Myc, prognosis, senescence, cisplatin

## Abstract

Patients with advanced bladder cancer gradually become less sensitive to chemotherapeutic agents, leading to tumor recurrence. Initiating the senescence program in solid tumors may be an important means of improving short-term drug sensitivity. The important role of c-Myc in bladder cancer cell senescence was determined using bioinformatics methods. The response to cisplatin chemotherapy in bladder cancer sample was analyzed according to the Genomics of Drug Sensitivity in Cancer database. Cell Counting Kit-8 assay, clone formation assay, and senescence-associated β-galactosidase staining were used to assess bladder cancer cell growth, senescence, and sensitivity to cisplatin, respectively. Western blot and immunoprecipitation were performed to understand the regulation of p21 by c-Myc/HSP90B1. Bioinformatic analysis showed that c-Myc, a cellular senescence gene, was significantly associated with bladder cancer prognosis and sensitivity to cisplatin chemotherapy. c-Myc and HSP90B1 expression were highly correlated in bladder cancer. Reducing the level of c-Myc significantly inhibited bladder cancer cell proliferation, promoted cellular senescence, and enhanced cisplatin chemosensitivity. Immunoprecipitation assays confirmed that HSP90B1 interacted with c-Myc. Western blot analysis showed that reducing the level of HSP90B1 could redeem the p21 overexpression caused by c-Myc overexpression. Further studies showed that reducing HSP90B1 expression could alleviate the rapid growth and accelerate cellular senescence of bladder cancer cells caused by c-Myc overexpression, and that reducing HSP90B1 levels could also improve cisplatin sensitivity in bladder cancer cells. HSP90B1/c-Myc interaction regulates the p21 signaling pathway, which affects cisplatin chemosensitivity by modulating bladder cancer cell senescence.

## INTRODUCTION

Bladder cancer is among the most common cancers worldwide and the seventh leading cause of cancer-related deaths. Histologically, bladder urothelial carcinoma (BLCA) is the most common bladder cancer subtype, accounting for 90% of all bladder cancers. Bladder cancer can be classified into four grades based on changes in the nuclei of cancer cells. The higher the grade, the worse the treatment outcome and prognosis. Treatment modalities for bladder cancer include radical surgery, neoadjuvant therapy, and conventional chemotherapy. The first-line treatment for bladder cancer is cisplatin-based combination chemotherapy [1]. Cisplatin-based chemotherapeutic regimens are considered promising; however, as the number of chemotherapy sessions increases, bladder cancer patients gradually become less sensitive to chemotherapeutic agents, leading to tumor recurrence and progression, in few cases death [2]. Even though decreased chemotherapy sensitivity in bladder cancer is a major factor in the poor prognosis of bladder cancer patients, the underlying molecular mechanisms are yet to be elucidated.

Cellular senescence is a state of growth arrest characterized by several phenotypic changes such as chromatin remodeling, reprogrammed metabolism, morphological changes, and upregulation of senescence-associated-galactosidase (SA-β-gal) activity [3]. In malignant tumors, cancer cells begin to proliferate uncontrollably, and somatic mutations are acquired more frequently than in normal tissues [4]. Thus, cellular senescence plays a key role in tumor suppression [5], by protecting tissues becoming cancerous through permanently arresting the cell cycle, inhibiting cell proliferation, and preventing the propagation of deleterious genetic mutations. For example, bladder [6], colorectal [7], and breast cancer cells [8] are subjected to permanent cell cycle arrest and inhibition of proliferation by inducing cellular senescence. Additionally, a growing number of studies suggest that initiating a senescence program in solid tumors may be an important means of improving drug sensitivity [9].

In the present study, 278 experimentally validated senescence-related genes were obtained from The Ageing Gene Database website [10]. By analyzing the expression, prognosis, and sensitivity to cisplatin chemotherapy of these genes in bladder cancer samples from The Cancer Genome Atlas (TCGA) database, c-Myc was identified as the best differential prognostic gene in bladder cancer and was most associated with cisplatin chemotherapy sensitivity. c-Myc is a prominent member of the MYC gene family. c-Myc is a transcription factor that regulates tumor cell cycle progression, proliferation, and apoptosis. Down-regulation of c-Myc inhibits self-renewal, tumorigenicity, invasiveness, and drug resistance of cancer stem cells in many malignancies [11]. Studies found that downregulation of c-Myc inhibits the growth and migration of cisplatin-resistant bladder cancer cells [12] and increases the susceptibility to cisplatin through reactive oxygen species-mediated apoptosis in human melanoma cells [13]. Previous studies have shown that c-Myc can induce tumor cell senescence by regulating downstream target genes in both breast [14] and liver cancer cells [15]. However, c-Myc regulation of cell senescence in bladder cancer has not been reported. Our experimental results demonstrated that the downregulation of c-Myc inhibited bladder cancer cell proliferation, promoted cell senescence, and increased cisplatin sensitivity. Furthermore, Heat shock protein 90 kDaβ1 (HSP90B1), a c-Myc-interacting gene, is strongly associated with cisplatin chemosensitivity in bladder cancer. In conclusion, our study demonstrated that HSP90B1 is involved in the regulation of the cellular senescence initiation gene p21 by c-Myc, a finding that provides new insights into improving cisplatin chemosensitivity in bladder cancer.

## RESULTS

### Differential senescence genes associated with cisplatin IC50 in bladder cancer

**Figure 1:**
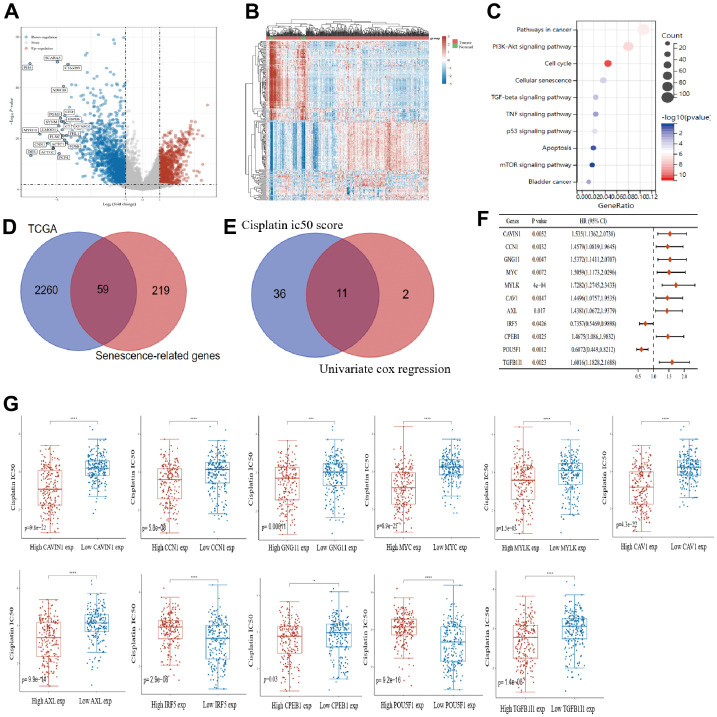
**Prognostic key genes of senescence associated with cisplatin IC50 in bladder cancer.** Volcano plot (**A**) and heat map (**B**) show the differential analysis of bladder cancer samples and normal bladder samples in The Cancer Genome Atlas (TCGA) database. (**C**) intersection of differential genes and senescence-associated genes in bladder cancer samples. (**D**) univariate analysis of senescence-associated differential genes and cisplatin IC50 score analysis. (**E**) univariate analysis results of 11 senescence genes were plotted using forest plots. (**F**) Correlations of 11 senescence genes were demonstrated using chord diagrams. (**G**) In bladder cancer, 11 senescence genes were analyzed in high and low expression groupings identify the differences between them and to compare cisplatin IC50 scores.

Differential expression analysis was performed on the 406 bladder cancer samples and 19 normal bladder tissue samples in TCGA database. The volcano plot (Figure 1A) and heat map (Figure 1B) show 2319 differential genes, of which 896 were upregulated and 1423 were downregulated. Kyoto Encyclopedia of Genes and Genomes (KEGG) enrichment analysis of the 2319 differential genes showed that they were significantly associated with cellular senescence in bladder cancer (Figure 1C). Based on the online Venn diagram tool, 278 senescence-associated genes downloaded from The Ageing Gene Database website were crosschecked with differential genes to obtain 59 senescence-associated differential genes (Figure 1D). Using these 59 senescence-related differential genes with high and low expression levels as groupings, a total of 47 genes had significant differences in cisplatin IC50 scores and among them, 13 prognostic genes were identified by one-way Cox regression analysis. Eleven senescence-related prognostic differential genes were obtained from the intersection set, and these were significantly associated with cisplatin IC50 scores (Figure 1E). The forest plot (Figure 1F) shows the prognostic characteristics of these 11 genes. Subsequently, we showed the differences in the cisplatin IC50 scores of these 11 key genes for senescence in bladder cancer samples grouped by high and low expression (Figure 1G).

### To construct senescence-related genes prognostic model

**Figure 2:**
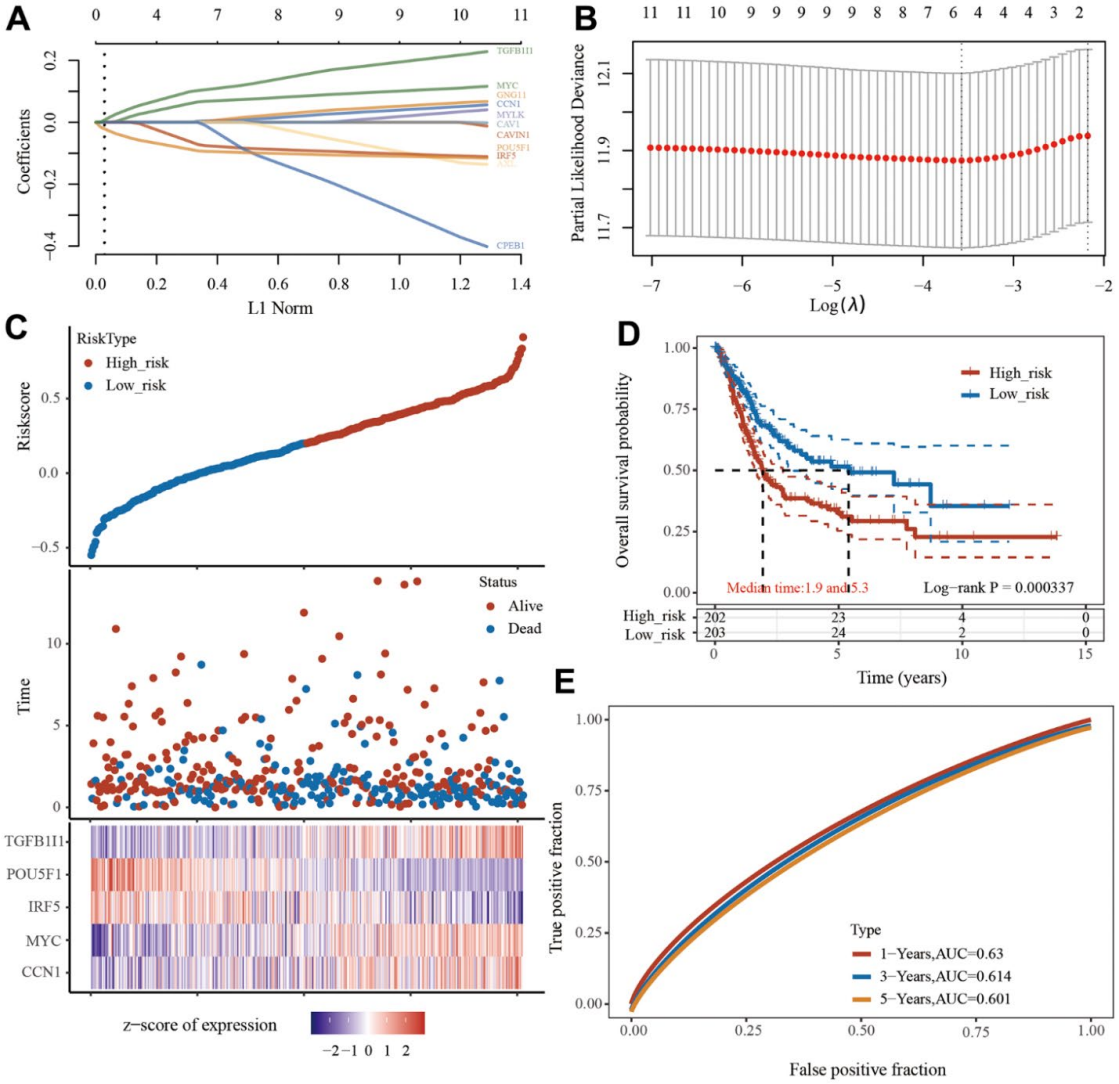
**Prognostic model of senescence-related genes.** (**A**) Partial likelihood deviance versus log (λ) was drawn using LASSO Cox regression model. (**B**) Coefficients of selected features are shown by lambda parameter. (**C**) Distribution of risk score, survival status, and the expression of 5 prognostic senescence-related genes in bladder urothelial carcinoma (BLCA). (**D**, **E**), Overall survival curves for BLCA patients in the high-/low-risk group and the ROC curve of measuring the predictive value.

The least absolute shrinkage and selection operator (LASSO) Cox regression model was used to select the most predictive genes as prognostic indicators, and their correlation with the cisplatin IC50 score was analyzed. λ was selected when the median of the sum of squared residuals was the smallest. Five potential predictors: *TGFB1I1*, *POU5F1*, *IRF5*, *MYC*, and *CCN1* were identified as prognostic factors for BLCA (Figure 2A, 2B). The risk score=(0.0016) *CCN1+(0.0635)*MYC+(-0.067)*IRF5+(-0.0898)*POU5F1+(0.1013)*TGFB1I1. The BLCA patients were divided into two groups based on their risk score. The distribution of the risk score, survival status, and expression of these five genes are shown in Figure 2C. Kaplan–Meier curves showed that patients with a high risk BLCA had a lower overall survival rate than that of patients with low risk BLCA (median time=1.9 and 5.3 years, p=0.000337) (Figure 2D). Finally, we also analyzed the predictive ability of this model for the survival of bladder cancer patients at one years, three years, and five years. The AUC values were 0.63, 0.614, 0.601 (Figure 2E). The higher the AUC value, the better the predictive power of the model [16].

### MYC identified as the best senescence-related prognostic gene in bladder cancer

**Figure 3:**
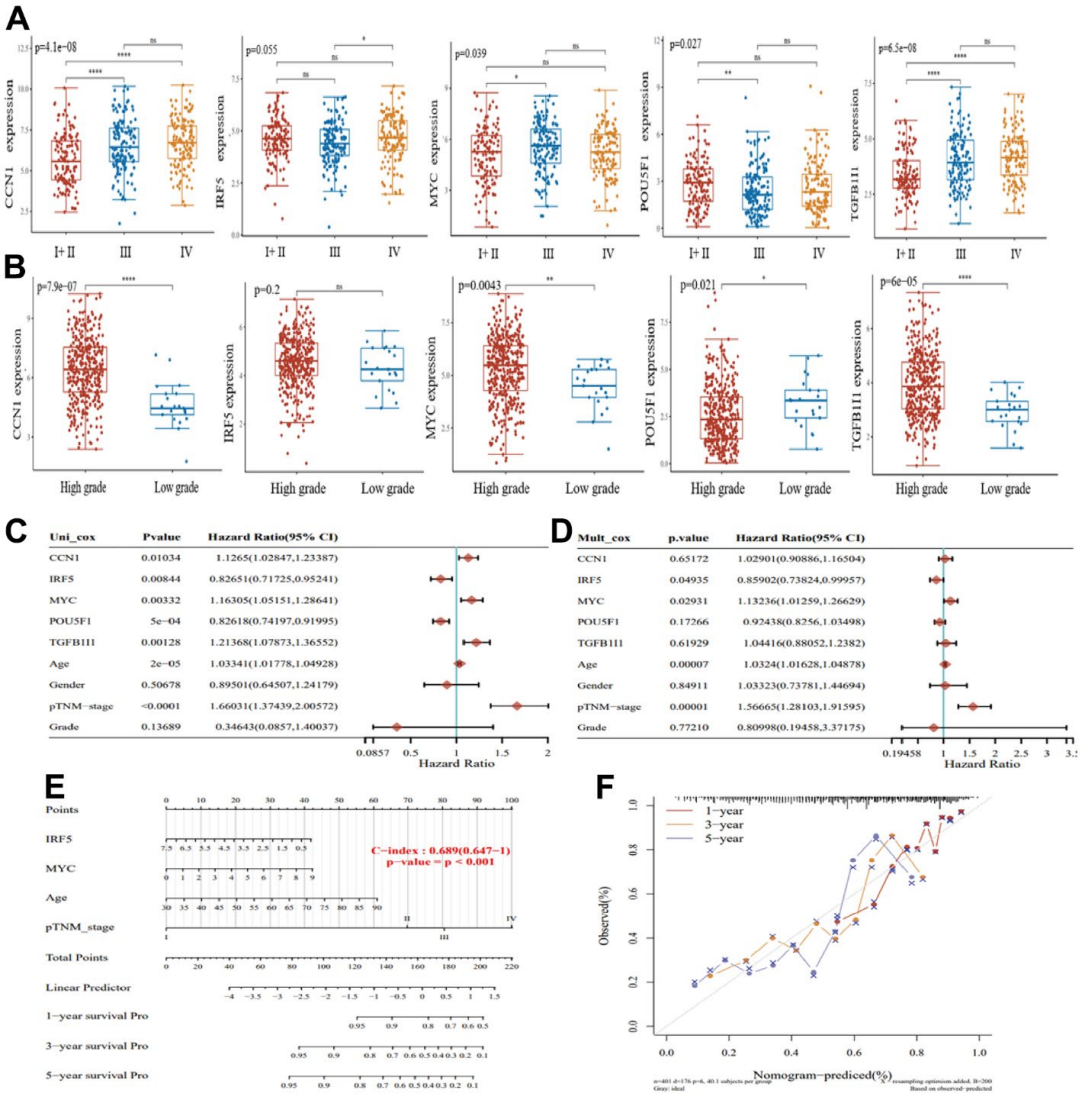
**Screening for the best senescence-related prognostic genes in bladder cancer.** (**A**) Expression of CCN1, IRF5, MYC, POU5F1, and TGFB1I1 in bladder cancer I+II (n=132), III (n=140), and IV (n=134). (**B**) Expression of CCN1, IRF5, MYC, POU5F1, and TGFB1I1 in bladder cancer high-grade samples (n=384) and low-grade samples (n=21). (**C**) Single-factor Cox regression analysis of prognostic differences among factors. (**D**) Multi-factor Cox regression analysis of prognostic differences among factors. (**E**) Columnar plots showing survival at 1, 3, and 5 years for the construct models. (**F**) Nomogram curves showing calibration curves for survival at 1, 3, and 5 years for the construct models.

Of the five genes obtained, we analyzed their expression in bladder cancer samples at different stages. *IRF5* (p=0.055), *CCN1* (p=4.1e-08), *MYC* (p=0.039), *POU5F1* (p=0.027), and *TGFB1I1* (p=6.5e-08) were significantly differentially expressed at different stages of bladder cancer (Figure 3A). Similarly, in TCGA database bladder cancer samples, *CCN1* (p=7.9e-07), *MYC* (p=0.0043), *POU5F1* (p=0.021), and *TGFB1I1* (p=6.0e-05) had significant differences in bladder cancer high-grade and low-grade samples, in addition to *IRF5* (p=0.2) (Figure 3B). To screen for the best senescence-related prognostic genes for bladder cancer, we included these five genes with clinicopathological parameters (age, sex, TNM stage, and grade) in univariate and multifactorial Cox regression models, and the results of multifactorial Cox regression analysis indicated that among these five genes, *IRF5* and *MYC* could be used as prognostic biomarkers in bladder cancer (Figure 3C, 3D). This result is visually illustrated by the column line plot (Figure 3E). We also show the calibration curves of this model for 1, 3, and 5-year survival using the nomogram curves (Figure 3F). We aimed to screen for the most valuable senescence-related genes in bladder cancer samples. The results of the multifactorial Cox regression analysis indicated that *c-Myc* and *IRF5* could be used as prognostic biomarkers for bladder cancer; however, we found no significant differences in IRF5 expression at different stages of bladder cancer. In summary, by analyzing the differential expression of the five genes in bladder cancer samples at different stages, we identified *MYC* as the best senescence-related prognostic gene for bladder cancer.

### Knockdown of c-Myc mediates cisplatin chemosensitivity through modulation of bladder cancer cell senescence

**Figure 4:**
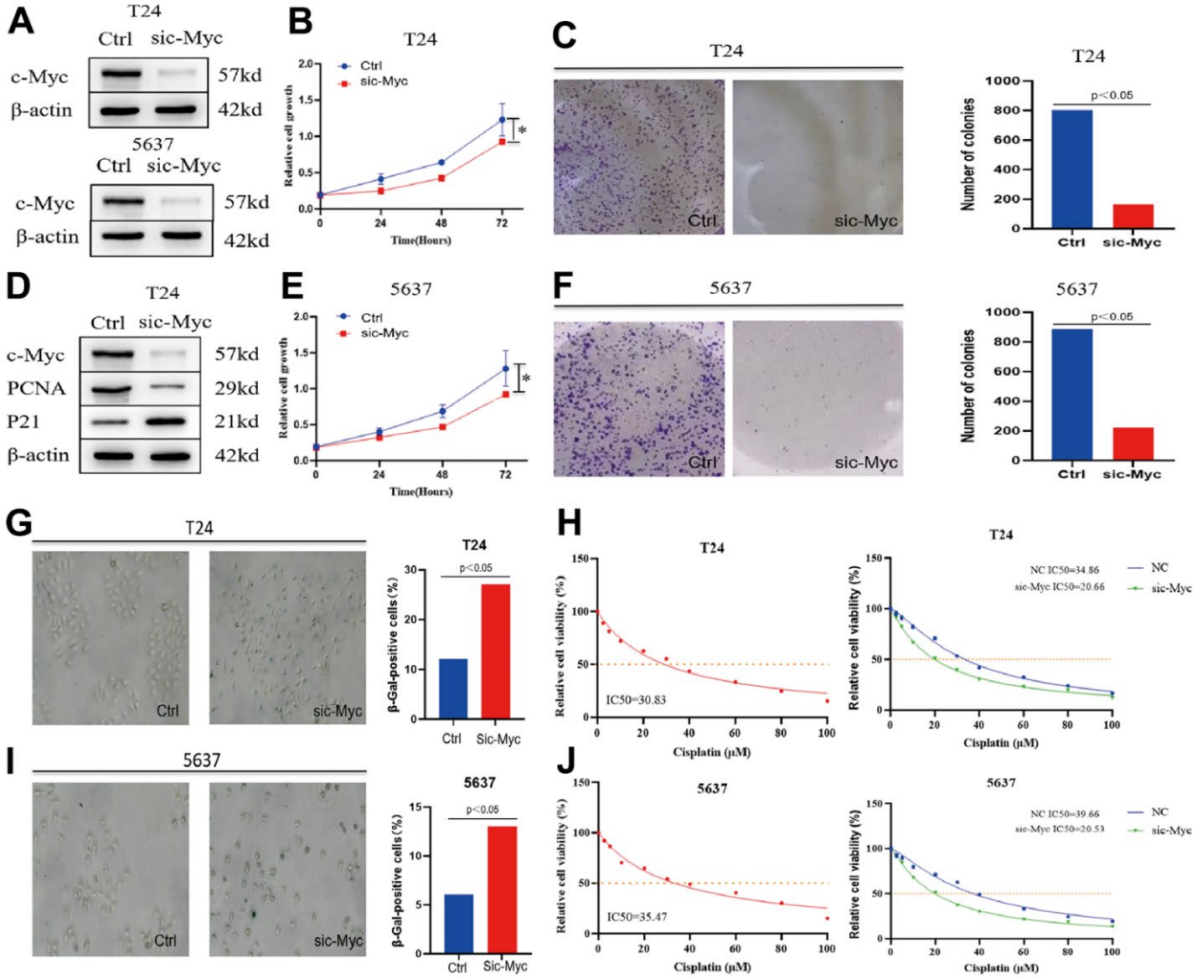
**Knockdown of c-Myc promotes bladder cancer cell senescence and affects the sensitivity of cisplatin chemotherapy.** (**A**) The expression of c-Myc protein was reduced in T24 and 5637 cells. (**B**, **C**) CCK8 and clonogenic assay was used to detect the effect of c-Myc knockdown on the proliferation of bladder cancer T24 cells. (**D**) The expression of c-Myc protein was decreased in T24 cells, and its effect on the expression of PCNA and P21 protein was detected. (**E**, **F**) CCK8 and clonogenic assay was used to detect the effect of c-Myc knockdown on the proliferation of bladder cancer 5637 cells. (**G**) c-Myc was knocked down in T24 cells, and its effect on cell senescence was analyzed by SA-β-gal. (**H**) c-Myc was knocked down in T24 cells to detect its effect on the sensitivity of cisplatin chemotherapy. (**I**) c-Myc was knocked down in 5637 cells, and its effect on cell senescence was analyzed by SA-β-gal. (**J**) c-Myc was knocked down in 5637 cells to detect its effect on the sensitivity of cisplatin chemotherapy.

Whether c-Myc regulates bladder cancer cell senescence has not yet been reported. To verify whether c-Myc directly regulates the proliferation of bladder cancer cells, we constructed a c-Myc- knockdown cell model by transfecting bladder cancer cells with c-Myc (Figure 4A). Clonogenic and CCK8 assay was performed to determine the effects of c-Myc on cell proliferation. The results revealed that reduction of c- Myc significantly slowed down cell growth compared to that in the control (Figure 4B, 4C). Microarray-based studies have indicated that p21 expression positively correlates with the expression of genes involved in cellular senescence [17]. Induction of p21 expression by a variety of stimuli is thought to drive senescence initiation [18]. Proliferating cell nuclear antigen (PCNA) is a key factor in DNA replication and cell cycle regulation [19]. In T24 cells, c-Myc increased p21 and decreased PCNA protein expression (Figure 4D). We repeated these experiments in the bladder cancer cell line 5637, and the results also proved that c-Myc can affect the proliferation of bladder cancer cells (Figure 4E, 4F). The level of SA-β-gal (a biomarker of senescence) was measured, and the percentage and strength of SA-β-gal-positive cells were significantly increased in c-Myc knockdown condition (Figure 4G–4I). To test whether c-Myc could influence cisplatin chemosensitivity in 5637 bladder cancer cells, we assayed the corresponding half-lethal dose (IC50) after cisplatin treatment. The IC50 was 30.83 μM in normal T24 cells and 35.47 μM in 5637 cells. We also found that the IC50 of cisplatin was significantly lower after the reduction of c-Myc expression (Figure 4H, 4J). Therefore, we believe that c-Myc regulates cisplatin chemosensitivity by affecting bladder cancer cell senescence.

### *HSP90B1* as a c-Myc interacting gene is highly associated with cisplatin sensitivity in bladder cancer

**Figure 5:**
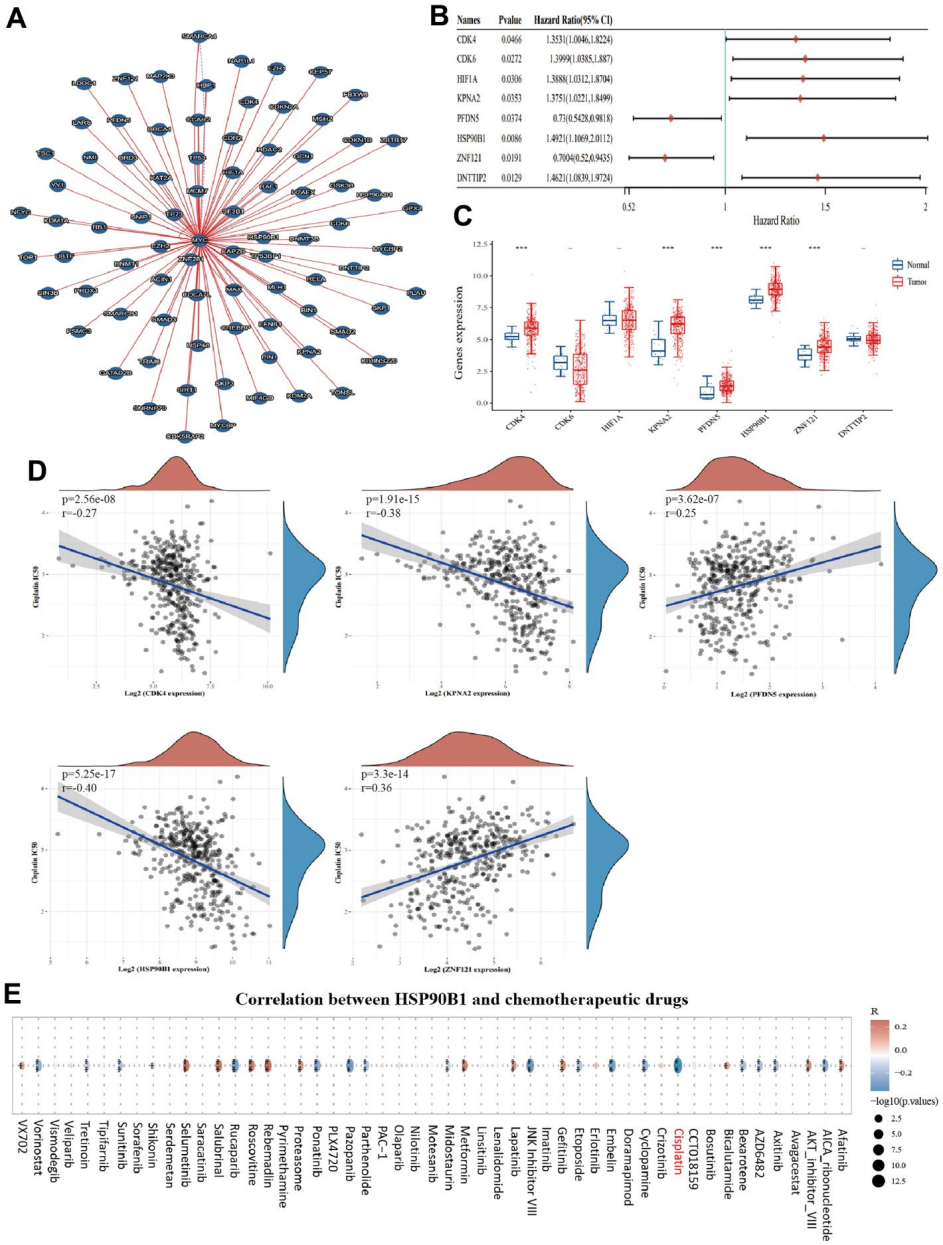
**HSP90B1 is significantly associated with cisplatin chemotherapy in bladder cancer.** (**A**) Interaction network map of c-Myc with other genes. (**B**) Forest plot of c-Myc interacting genes with prognostic significance in bladder cancer. (**C**) c-Myc interacting genes with prognostic value expressed in bladder cancer and normal bladder tissues. (**D**) Five key genes with cisplatin IC50 score for correlation. (**E**) Correlation of HSP90B1 with chemotherapeutic agents in bladder cancer.

**Figure 6:**
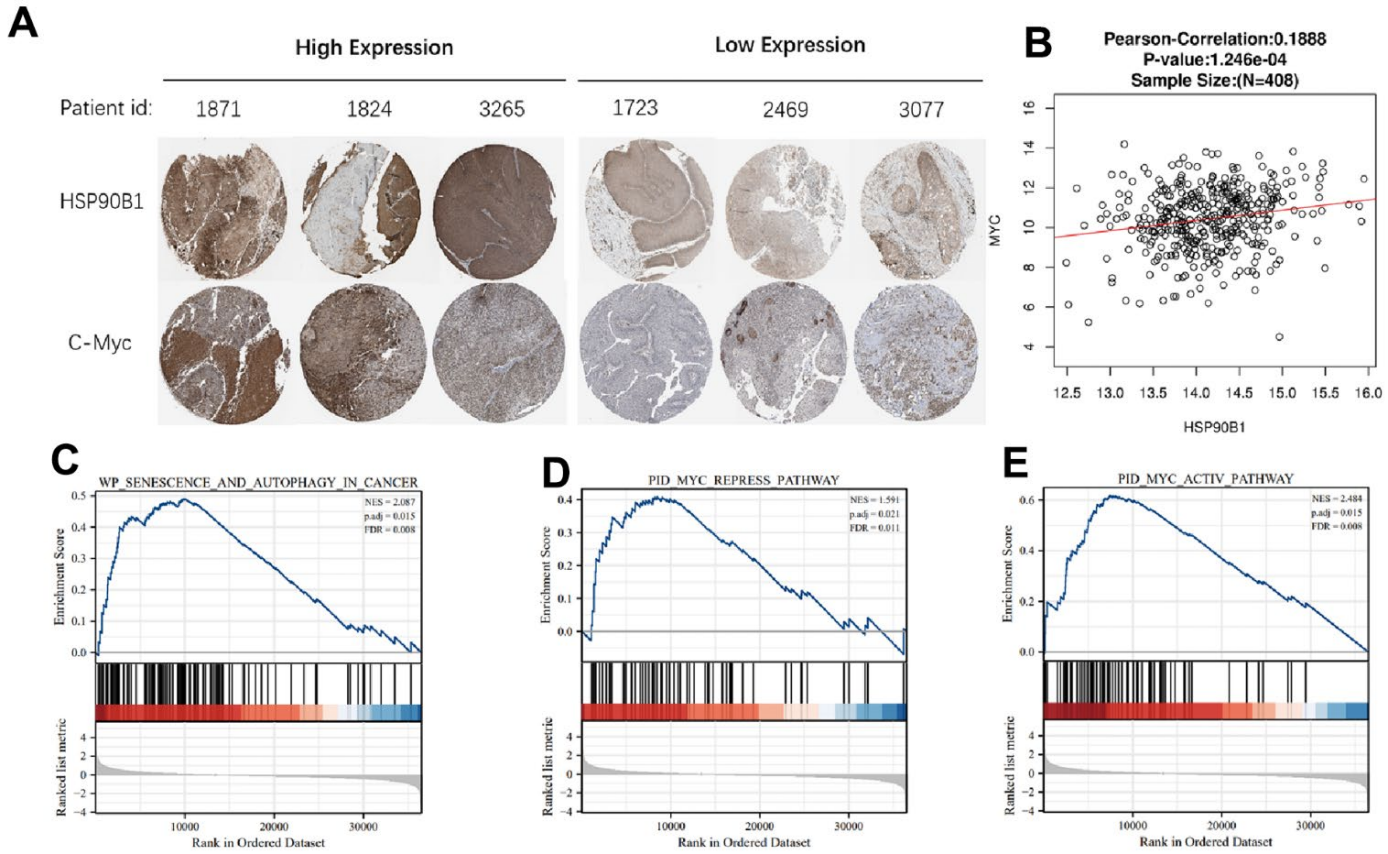
**HSP90B1 and c-Myc are highly correlated.** (**A**) Expression of HSP90B1 and c-Myc protein in bladder cancer. (**B**) Expression of HSP90B1 and c-Myc mRNA in bladder cancer samples. (**C**–**E**) Gene enrichment analysis of HSP90B1 in bladder cancer.

To explore the possible mechanisms by which c-Myc regulates bladder cancer cell senescence, we identified 79 proteins that interacted with c-Myc (direct and indirect evidence of interaction with c-Myc) using the Molecular Interaction Search Tool (MIST) (Supplementary Table 1). As shown in Figure 5A, we mapped the interaction network of *c-Myc* with other genes. Subsequently, we analyzed the expression and prognostic value of these 79 genes in bladder cancer samples from TCGA database. *CDK4*, *KPNA2*, *PFDN5*, *HSP90B1*, and *ZNF121* were identified as prognostic differential genes in bladder cancer (Figure 5B, 5C). Among these five genes, *HSP90B1* was strongly associated with cisplatin chemosensitivity (Figure 5D). Furthermore, to demonstrate the important role of *HSP90B1* in bladder cancer chemotherapy, we analyzed the correlation between *HSP90B1* and all chemotherapeutic agents in bladder cancer, and the results showed that *HSP90B1* was strongly associated with cisplatin (Figure 6E). Thus, among other genes that interact with c-Myc, we suggest that HSP90B1 plays an important role in cisplatin chemotherapy in bladder cancer and may be involved in the regulation of bladder cancer cell senescence by c-Myc.

### HSP90B1 is highly associated with c-Myc and is involved in the regulation of bladder cancer cell senescence

To further analyze the relationship between HSP90B1 and c-Myc in bladder cancer, we analyzed the expression of HSP90B1 and c-Myc proteins in six bladder cancer samples selected by The Human Protein Atlas and found that HSP90B1 and c-Myc protein expression was significantly correlated (Figure 6A). In TCGA database bladder cancer samples, the mRNA expression of HSP90B1 and c-Myc mRNA expression were also significantly correlated (Figure 6B). The results of HSP90B1 gene enrichment analysis also showed that HSP90B1 was highly correlated with c-Myc and that HSP90B1 could regulate bladder cancer cell senescence in bladder cancer samples from TCGA database (Figure 6C–6E). Therefore, we suggest that HSP90B1 is involved in the regulation of bladder cancer cell senescence via its interaction with c-Myc.

### HSP90B1 interacts with c-Myc to affect bladder cancer cell senescence by regulating *p21*

**Figure 7:**
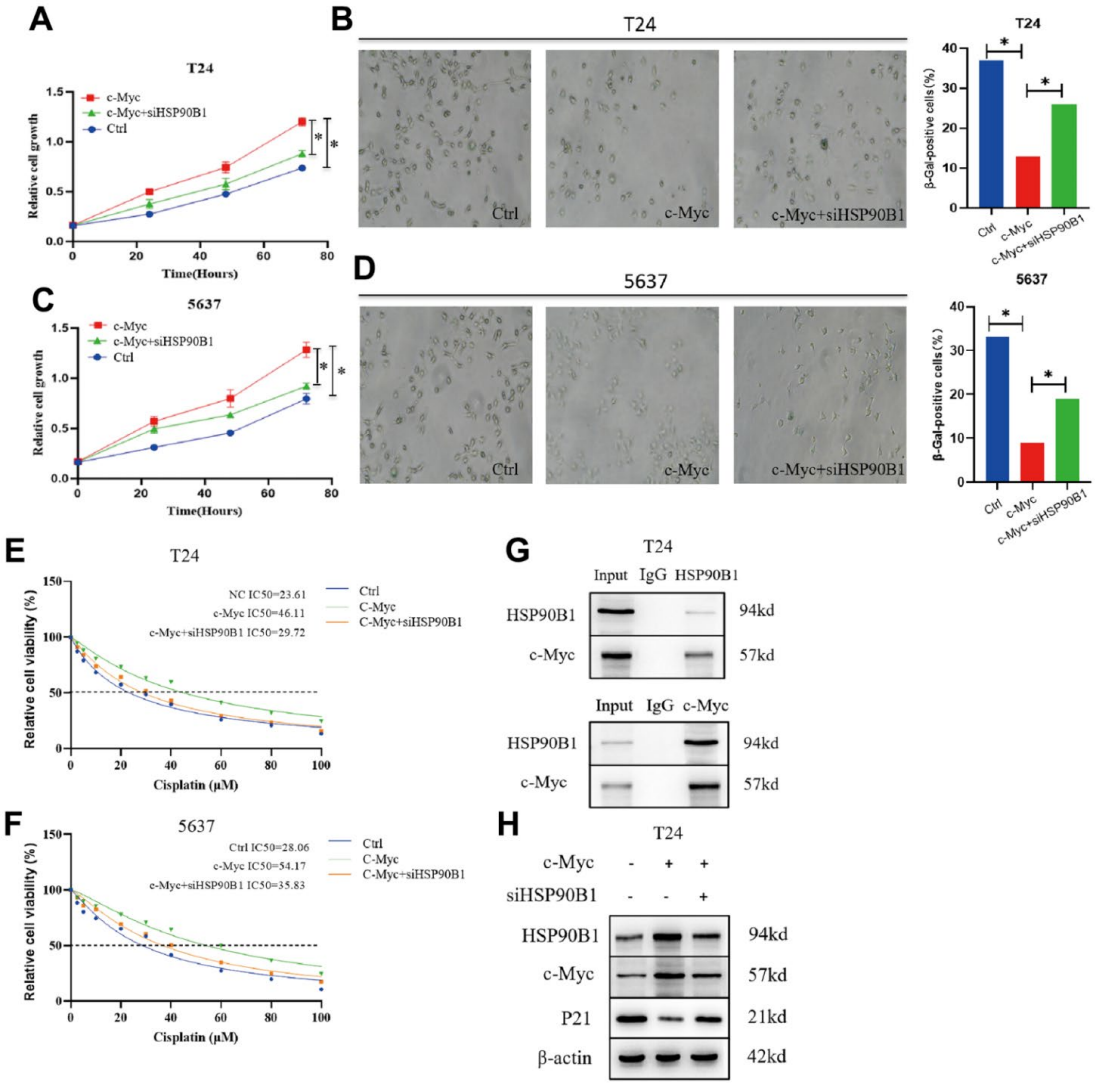
**c-Myc/HSP90B1 interaction regulates cisplatin sensitivity by affecting bladder cancer cell senescence.** (**A**) Knockdown of HSP90B1 to analyze its effect on c-Myc regulation of T24 cells proliferation ability. (**B**) Knockdown ofHSP90B1 to analyze its effect on c-Myc regulation of T24 cells senescence. (**C**) Knockdown of HSP90B1 to analyze its effect on c-Myc regulation of 5637 cells proliferation ability. (**D**) Knockdown of HSP90B1 to analyze its effect on c-Myc regulation of 5637 cells senescence. (**E**, **F**) Knockdown of HSP90B1 to analyze its effect on c-Myc-regulated cisplatin sensitivity in T24 and 5637 cells. (**G**) CO--IP analysis with HSP90B1 or c-Myc antibody in T24 cells. (**H**) Western blot analysis of the effect of HSP90B1 on c-Myc-regulated p21 signaling pathway.

From the results of the above bioinformatics analysis, we conclude that HSP90B1, a c-Myc-interacting gene, plays an important role in bladder cancer cell senescence. CCK8 experiments showed that overexpression of *c-Myc* significantly enhanced the proliferation of bladder cancer cells (T24 and 5637), while knockdown of *HSP90B1* reversed this result (Figure 7A–7C). In addition, we found that overexpression of *c-Myc* significantly reduced the number of senescent cells in bladder cancer cells (T24 and 5637), while knockdown of *HSP90B1* restored senescence in bladder cancer cells (Figure 7B, 7D). To determine whether HSP90B1 interacts with c-Myc, we performed a Co-IP assay (Figure 7G). Finally, we analyzed whether HSP90B1 is involved in the regulation of *p21* by c-Myc by overexpressing c-Myc protein. We found that the expression of both HSP90B1 and c-Myc proteins was significantly enhanced, and that of p21 protein was significantly reduced. The knockdown of HSP90B1 restored the inhibitory effect of c-Myc on p21 (Figure 7H). Therefore, we suggest that HSP90B1 is involved in the regulation of the p21 signaling pathway by c-Myc.

## DISCUSSION

Owing to multiple endogenous and exogenous stressors, damage to cells can sometimes persist despite the existence of wide range of repair mechanisms. Replicating cells also require defense mechanisms to reduce the effects of these persistent stressors on tissue degeneration and limit cancer progression. One of these defense mechanisms involves switching to a stable state of survival with reduced proliferative capacity, known as cellular senescence [20]. Cellular senescence serves as an important mechanism, second only to apoptosis, to eliminate damaged cells during physiological and pathological processes and maintain tissue homeostasis [21]. Thus, in many cases, cellular senescence is a protective process that protects tissues from cancer development by permanently arresting the cell cycle, inhibiting proliferation, and preventing the propagation of deleterious genetic mutations. To summarize, cellular senescence acts as a barrier to tumorigenesis, leading to tumor arrest or regression by preventing cancer cell proliferation. The cell cycle protein-dependent kinase inhibitor p21 (CDKN1A or p21WAF1/Cip1), a member of the Cip/Kip family, plays a key role in regulating the cell cycle and cellular senescence by blocking the activity of cell cycle protein-dependent kinases (CDK), including CDK1 and CDK2 [22, 23].

To further explore the role of cellular senescence in bladder tumorigenesis, we performed a series of experiments in bladder cancer cell lines and found that c-Myc knockdown promoted bladder cancer cell senescence. The transcription factor *c-Myc* is an important oncogene, and overexpression of *c-Myc* is associated with tumorigenesis and cell proliferation [24, 25]. c-Myc regulates the expression of cyclin D1, cyclin E, and *p21*, which in turn promotes cell movement from G1 to S phase and, ultimately, cell growth. The role of c-Myc in bladder cancer has been well documented. c-Myc can regulate the growth, proliferation, and metastasis of bladder cancer cells [26–28], and in addition, lowering c-Myc levels can reduce the resistance of bladder cancer cells to cisplatin [29]. c-Myc has been shown to be closely related to cellular senescence, affecting the transcription of senescence-related genes, such as *p16*, *p53*, and *p21*, as well as regulating telomerase activity. In breast cancer, downregulation of c-Myc can promote senescence in breast cancer cells by activating Rb1/p21 [14], while in liver cancer, c-Myc can induce senescence in hepatocellular carcinoma cells by regulating telomerase activity [15]. As a key marker of cellular senescence, p21 has been shown to initiate the onset of senescence. Various stimuli, including mitochondrial dysfunction, radiotherapy, and chemotherapeutic drugs, induce cellular senescence through the DNA damage response by activating the ataxia telangiectasia-mutated (ATM) gene and ultimately the p53/p21 pathway [30]. Although *p21* is mainly regulated by p53 at the transcriptional level, multiple transcription factors (BRCA1, Smad3, AP4 and c-Myc) have also been reported to control transcriptional activation or repression of *p21* [31–33]. In our study, we demonstrated for the first time that c-Myc knockdown increased *p21* expression that promoted bladder cancer cell senescence, thus affecting cisplatin sensitivity.

To explore the possible molecular mechanisms of c-Myc-mediated regulation of p21, we screened 79 genes interacting with c-Myc using the MIST database. Among the c-Myc-interacting genes, *HSP90B1* showed significantly differential expression in bladder cancer compared to its expression in corresponding normal tissues and showed the highest correlation with the cisplatin IC50 score. HSP90B1 is a stress-induced molecular chaperone and a member of the heat shock protein (HSP) 90 family, also known as GRP94 and GP96 [34, 35], which is normally expressed at low levels to control the cell cycle during cell growth and development. HSP90B1 plays a central role in regulating the correct folding, stability and function of many client proteins [36]. Inhibition of HSP90 activity leads to the aggregation or proteasomal degradation of these clients, which in turn promotes the disruption of numerous oncogenic signaling pathways essential for tumor cell proliferation and survival [37]. In ovarian cancer cells, inhibition of HSP90 levels reversed cisplatin resistance [38]. Based on cancer stem cell theory, cancer stemness is enhanced in surviving tumor cells, which are referred to as initiating cells, after cisplatin chemotherapy, and HSP90 inhibitors improve the sensitivity of bladder cancer initiating cells to cisplatin chemotherapy [39]. HSP90 is closely related to chemoresistance, therefore, we hypothesized that HSP90B1, a member of the HSP90 family, has an important role in chemoresistance.

In addition, experimental studies have demonstrated a relationship between HSP90 and cellular senescence. The inhibition of HSP90 expression induces senescence in lung cancer cells [40], and HSP90β inhibits senescence during skeletal muscle regeneration [41]. In highly chemo-resistant malignant pleural mesothelioma, the chaperone protein HSP90 plays an important role in assisting the chemotherapy-induced senescence-associated secretory phenotype, and inhibition of HSP90 can modulate chemotherapy sensitivity by reducing IL-8 levels [42]. Our previous findings suggest that HSP90B1 is highly expressed in bladder cancer and can be used as a prognostic biomarker in patients with bladder cancer [43]. Additionally, HSP90B1 regulates the growth and invasiveness of bladder cancer cells [44]. In the present study, we demonstrated that HSP90B1 regulates p21 expression by enhancing the function of c-Myc through protein-protein interactions, and that increasing the expression of HSP90B1 can alleviate bladder cancer cell senescence caused by the knockdown of c-Myc.

Our findings suggest that c-Myc promotes bladder cancer cell senescence by regulating the p21 signaling pathway to improve cisplatin chemosensitivity. HSP90B1 interacts with c-Myc in bladder cancer and is involved in the regulation of bladder cancer cell senescence by c-Myc. In conclusion, our study provides new insights for improving cisplatin sensitivity in bladder cancer.

## MATERIALS AND METHODS

### Sample and data

The Genomic Data Commons (GDC) portal of TCGA database (https://portal.gdc.cancer.gov/) was used to obtain clinical information on BLCA patients, including 406 BLCA tissue samples and 19 normal bladder samples. The Aging Gene Database (https://genomics.senescence.info/genes/index.html) and cBioPortal database (https://www.cbioportal.org) were used to obtain the mutation information of 278 experimentally validated senescence-associated genes. MIST (https://fgrtools.hms.harvard.edu/MIST/) was used to identify 79 proteins interacting with c-Myc. The expression of *c-Myc* and *HSP90B1* in bladder cancer was obtained from The Human Protein Atlas database (https://www.proteinatlas.org/).

### Antibodies and reagents

c-Myc (ab32072), HSP90B1 (ab238126), p21 (ab108349) and PCNA (ab92552) were purchased from Abcam. Actin (81115-1-RR) was purchased from ProteinTech.

### Cisplatin sensitivity analysis and gene enrichment analysis

Genomics of Drug Sensitivity in Cancer (GDSC) (https://www.cancerrxgene.org/) was used to predict the chemotherapy response for each bladder cancer sample. The prediction process was implemented using the R package pRRophetic, where the half-maximal inhibitory concentration (IC50) of samples was estimated by ridge regression; all parameters were set at default values using batch effects of combat and tissue type, and duplicate gene expression was summarized as the mean. Sample significance for both groups was tested using the Wilcoxon test: *p<0.05, **p<0.01, and ***p<0.001.

Enrichment analysis is useful for analyzing gene functions and related high-level genomic functional information. To better understand the oncogenic role of the target genes, the ClusterProfiler package in R was used to perform Gene Set Enrichment Analysis (GSEA) and KEGG signaling pathway analysis.

### Construction of prognostic models

The LASSO regression algorithm was used for feature selection and 10-fold cross-validation, while log rank was used to test Kaplan–Meier survival analysis comparing survival differences between two or more groups, as described above. Time ROC analysis was performed to determine the accuracy of the prediction model. For the Kaplan-Meier curves, p-values and hazard ratios (HR) with 95% confidence intervals (CI) were derived using the log-rank test and univariate Cox regression. Univariate and multivariate Cox regression analyses were performed using the “forestplot” package, and the forest plots showed each variable (p-value, HR and 95% CI). Based on the results of multivariate Cox proportional risk analysis, column line plots were created using the “rms” package to predict the total recurrence rate at 1, 3, and 5 years. The line graphs provide graphical results for these factors and allow calculation of the prognostic risk of individual patients based on the points associated with each risk factor.

### Cell proliferation ability assay

Cell proliferation capacity was determined using the CCK8 assay. Bladder cancer cells were inoculated into 96-well plates (5 × 10^3^ cells/well) and treated according to the appropriate procedure, with or without siRNA transfection of tumor cells. CCK8 reagent was added to the wells at 2 h (data at 0 h categorized in the experiment), 24 h, 48 h, and 72 h after plate laying. The absorbance of each well was measured at 450 nm (A450) using a microplate reader. Cell proliferation capacity was determined using clone formation assay. Tumor cells transfected with or without siRNA, and bladder cancer cells were seeded into 6-well plates (500 cells/well). After cloning, the cells were washed once with PBS, followed by addition of 2 mL of 4% paraformaldehyde to each well for 15-30 min, and washed once again with PBS; then, 2 ml of crystal violet staining solution was added to each well and stained for 20-30 min. Finally, the cells were washed once more with PBS and air-dried for imaging.

### Senescent cell assay

Senescent cells were measured using β-galactosidase staining (Senescence β-Galactosidase Staining Kit). Bladder cancer cells were inoculated into 6-well plates and stained with freshly prepared SA-β-Gal staining solution according to the manufacturer’s instructions (Biyuntian Biotechnology Co., Ltd., Shanghai, China). Stained cells were examined through a microscope and the percentage of senescent cells was quantified by calculating the percentage of SA-β-Gal positive cells in randomly selected areas (n=3).

### Western blot analysis and immunoprecipitation

Bladder cancer cells were collected, washed twice with cold PBS, and lysed in NP-40 lysis buffer for 30 min at 4° C. The protein concentrations were measured using a bicinchoninic acid assay kit (Thermo Fisher Scientific Inc.). Protein extracts were separated by electrophoresis on 8-12% precast sodium dodecyl sulfate-polyacrylamide microgels (Tris-HCl SDS-PAGE) and transferred to polyvinylidene difluoride membranes. The membranes were incubated with the indicated antibodies and detected using chemiluminescence. For immunoprecipitation, total cell lysates were incubated with appropriate antibodies overnight and then rotated with protein A/G beads for 2 h to 4 h at 4° C. The beads were washed three times with NP-40 lysis buffer with SDS sample buffer and boiled for 5-10 min. Co-precipitation was analyzed using Western blot.

### Statistical analysis

The significance of the expression of senescence-associated genes in TCGA database at different staging levels and the distribution of IC50 scores passed the Wilcoxon test. Differential expression of senescence-related genes in bladder cancer samples from the stage-graded TCGA database was determined using the Kruskal–Wallis test. Statistical analyses were performed using R software v4.0.3 (R Foundation for Statistical Computing, Vienna, Austria). Statistical significance was set at p < 0.05.

### Availability of data and materials

The datasets generated and/or analyzed in the current study are available from the corresponding author upon reasonable request.

## Supplementary Material

Supplementary Table 1
